# Analytical Evaluation of Simulated Kidney-Disease Profiles: Comparison of a Portable Near-Infrared Spectrophotometer with a Benchtop FT-NIR

**DOI:** 10.3390/bios16100573

**Published:** 2026-10-12

**Authors:** Diogo Serrano, Paulo Zoio, Bernardo B. Carvalho, Luís P. Fonseca, Cecília R. C. Calado

**Affiliations:** 1 iBB—Institute for Bioengineering and Biosciences, i4HB—The Associate Laboratory Institute for Health and Bioeconomy, IST—Instituto Superior Técnico, Universidade de Lisboa, 1049-001 Lisbon, Portugalpjzoio@gmail.com (P.Z.); luis.fonseca@tecnico.ulisboa.pt (L.P.F.); 2 ISEL—Instituto Superior de Engenharia de Lisboa, Instituto Politécnico de Lisboa, 1549-020 Lisbon, Portugal; 3 Instituto de Plasmas e Fusão Nuclear, IST—Instituto Superior Técnico, Universidade de Lisboa, 1049-001 Lisboa, Portugal; bernardo.carvalho@tecnico.ulisboa.pt

**Keywords:** kidney diseases, IR spectroscopy, biomarkers

## Abstract

Chronic kidney disease (CKD) is commonly assessed using blood biomarkers such as creatinine, urea and albumin. This study compared a portable six-channel near-infrared (NIR) analyzer (miniNIR; 1300–2100 nm) with a benchtop Fourier-transform near-infrared (FT-NIR) instrument for the discrimination and quantification of fifty-four independent concentration-defined profiles in composed fetal bovine serum solutions spanning six simulated KDIGO categories. SVM-RBF, Random Forest, XGBoost, Logistic regression and partial least-squares discriminant analysis (PLS-DA) were evaluated. For miniNIR binary ‘healthy/disease’ profile discrimination, SVM-RBF yielded a validation AUC of 1.00, accuracy of 0.91, sensitivity of 1.00 and specificity of 0.83. The six-class profile assignment was substantially more difficult: PLS-DA was selected by grouped nested cross-validation, but validation accuracy was 0.18, macro-F1 was 0.11 and macro-AUC was 0.77 across the evaluable classes. For miniNIR regression, validation R^2^ values were 0.48 for creatinine, 0.30 for urea and 0.10 for albumin. The strongest FT-NIR validation results were R^2^ = 0.64 for creatinine, 0.79 for urea and 0.89 for albumin, depending on the spectral window. For FT-NIR binary ‘healthy/disease’ profile discrimination, SVM-RBF yielded a validation AUC of 0.99, accuracy of 0.71 and a specificity of 0.47. Although the high binary AUC in this controlled concentration design for the miniNIR, it should not be interpreted as evidence of CKD-specific recognition or clinical performance. The comparison quantifies the information retained and lost when continuous FT-NIR sampling is reduced to six broad discrete channels; validation in clinically characterized human samples is required before any screening application can be considered.

## 1. Introduction

Fourier-transform infrared (FTIR) spectroscopy has become a powerful, label-free tool for the analysis of biofluids in medical diagnosis, offering high molecular specificity, minimal sample preparation, rapid measurement, low operational cost, and the simultaneous readout of several biomolecular classes [1,2]. The mid-infrared (MIR) region, which probes the fundamental vibrational modes of biomolecules, provides particularly high chemical specificity for biomarker discovery and clinical screening [1]. Its main limitation for biofluids is the strong absorption of water, which can mask analytically relevant bands and often requires additional steps, such as sample dehydration [2]. Near-infrared (NIR) spectroscopy circumvents several of these constraints. Although NIR bands arise from overtones and combination vibrations and are therefore intrinsically broader and less chemically specific than MIR spectral bands, NIR radiation is far less absorbed by water and penetrates deeper into hydrated matrices, making it well suited to the direct analysis of aqueous biofluids [2,3]. Consequently, NIR measurements can frequently be performed on native biofluids with little or no preparation, while retaining short acquisition times and, importantly, compatibility with compact and low-cost instrumentation [4]. These features make NIR especially attractive for point-of-care (POC) and decentralized clinical applications.

Interest in applying vibrational spectroscopy to kidney-disease diagnosis has grown steadily, with studies detecting Chronic Kidney Disease (CKD)-associated biochemical alterations in serum, plasma, urine, and saliva [2]. Using attenuated total reflectance Fourier-transform mid-infrared (ATR-FT-MIR) spectroscopy, Tangwanichgapong et al. [5,6] identified serum molecular biomarkers that discriminate CKD from controls and demonstrated the potential of this approach for POC screening in saliva. More recently, NIR spectroscopy has been applied to the analysis of salivary in other chronic diseases [7]. On the NIR side, several works have targeted the quantification of urea and creatinine—the cornerstone indicators of kidney function—directly in biofluids [8,9]. In our own recent work, we showed that both benchtop FT-MIR and FT-NIR spectroscopy can predict creatinine, urea, and albumin in fetal-bovine-serum solutions designed to reproduce coordinated biochemical variation across simulated CKD categories [10,11]. Those controlled studies established analytical feasibility in composed solutions but did not establish disease-specific signatures or clinical performance.

Translating spectroscopic renal diagnostics into routine practice requires instrumentation that is portable, affordable and operable outside specialized laboratories, in addition to benchtop equipment. Portable NIR spectrometers are attractive because they combine the intrinsic advantages of NIR with small size, low power consumption, short analysis times, and real-time measurement at POC [4,12], enabling deployment in outpatient clinics, primary care, dialysis units, or home-monitoring settings. Their analytical maturity is now well documented: low-cost handheld devices have achieved reliable classification and quantification in demanding real-world settings, from the authentication and quality control of food and feed [13] to the identification of organic solvents and the low-level quantification of water contamination in industry, where a handheld sensor was benchmarked directly against a benchtop FT-NIR instrument [14]. In the clinical domain, portable NIR methods are under investigation for point-of-care applications, including infectious-disease testing. Current evidence nevertheless supports treating NIR as an investigational analytical or screening approach rather than a definitive diagnostic method [15].

Particularly relevant to renal applications is the work of Suzuki et al. [16], who used a Light-emitting diode (LED)-based NIR system with discrete diode sources for reagent-free estimation of urea and creatinine in spot urine, demonstrating the feasibility of miniaturized, LED-driven spectrometers for quantitative analysis in turbid biofluids; earlier NIR studies in urine similarly quantified urea, creatinine, and protein with high accuracy [17,18]. Table 1 summarizes representative benchtop, portable and point-of-care spectroscopic approaches proposed for renal-biomarker analysis, showing that most have addressed urinary biomarkers or non-NIR modalities, and situating the present comparison of a portable NIR device against a benchtop FT-NIR for simulated-serum CKD analysis.

The portable device evaluated in the current work uses six commercially available LED channels spanning nominal wavelengths of 1300–2100 nm. The channels were selected from the manufacturer’s catalogue within the target range and were not derived by a formal wavelength-optimization procedure. Their broad, partly overlapping emission bands sample portions of N–H, O–H and C–H overtone and combination regions, while reducing size, power demand and data dimensionality relative to continuous-spectrum FT-NIR. The same integrated platform was previously applied by our group in an industrial comparison with a benchtop FT-NIR reference [20]. In the present study, both instruments were evaluated using the same grouped regression and classification workflow.

The objective of this study was to quantify how much analytical information is retained when continuous, high-resolution FT-NIR sampling is replaced by a portable six-channel NIR analyzer in composed serum solutions with concentration-defined profiles. Specifically, we assessed the ability of both platforms to: (i) discriminate healthy-like from CKD-like formulations; (ii) assign formulations to simulated KDIGO categories; and (iii) predict creatinine, urea and albumin concentrations. This controlled comparison extends our preceding MIR–NIR studies [10,11] by introducing substantial spectral miniaturization, while providing an initial analytical feasibility assessment rather than clinical validation of CKD screening or staging.

## 2. Materials and Methods

### 2.1. Sample Preparation

A set of 54 composed fetal bovine serum solutions was prepared as described in [11]. In brief, fetal bovine serum (FBS) diluted 1:5 (*v*/*v*) in ultrapure water was used as a common matrix and spiked with creatinine, urea, and albumin to simultaneously reproduce the biochemical profiles of the six Kidney Disease Improving Global Outcomes (KDIGO) categories of CKD, with nine independent solutions per category. Each formulation generated five FT-NIR replicate spectra and ten miniNIR replicate readings, these being instrumental replicates treated as repeated measurements of the same experimental unit. To enable a direct instrument comparison, every solution was measured under the same conditions as both the benchtop FT-NIR spectrometer and the miniNIR prototype, so that the two spectral datasets differ only in the acquisition device.

### 2.2. Infrared Spectroscopy

The benchtop FT-NIR spectroscopic analysis was conducted as described by Sales et al. [9] and Monteiro et al. [20]. Spectra were acquired using an NIR transflection fiber optic probe IN-271P (Bruker Optics, Billerica, MA, USA), with a path length of 2 mm, coupled to a Vertex-70 spectrometer (Bruker Optics). Background signal removal (blank) of the atmosphere was done before every sample read. Spectra were collected in the 12,500–4000 cm^−1^ range, consisting of 32 co-added scans with 8 cm^−1^ resolution. The scanner velocity was set to 10 kHz, and the aperture setting was defined as 6 mm.

**Figure 1:**
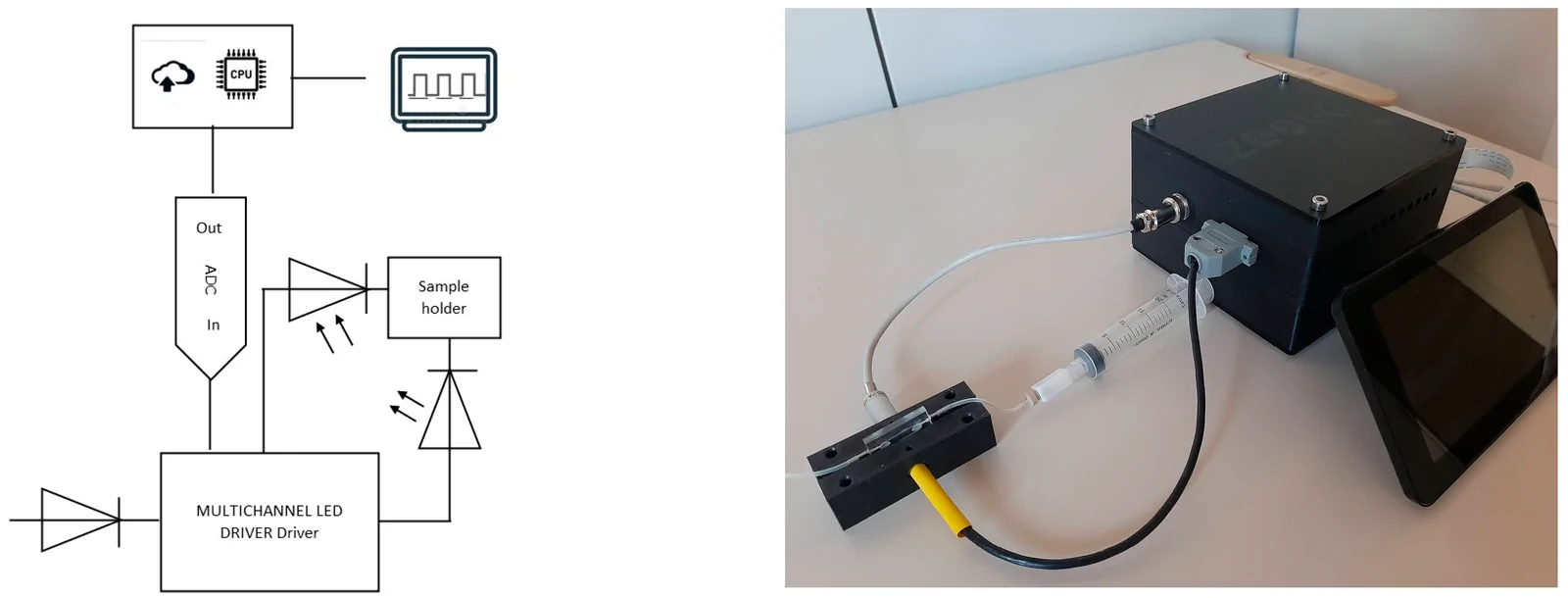
Schematic representation of the miniNIR, a prototype of a portable miniaturized NIR spectrometer and an image highlighting the flow cuvette.

An in-house assembled NIR prototype spectrometer, miniNIR, was employed, operating in the spectral range of 1300–2100 nm (7692–4762 cm^−1^), which covers the first overtone and combination regions of the N–H, O–H, and C–H vibrations associated. The miniNIR device is powered via a standard 5 V USB interface and is fully controlled by computer software, facilitating portability and integration into point-of-care workflows. It uses a matrix of six commercial LED (Microsensor NT LLC, St. Petersburg, Russia) centered at 1300 nm (7692 cm^−1^), 1400 nm (7143 cm^−1^), 1600 nm (6250 cm^−1^), 1700 nm (5882 cm^−1^), 1900 nm (5263 cm^−1^), and 2100 nm (4762 cm^−1^). This specific configuration allows selective illumination of key spectral windows that contain overtone and combination bands relevant to the target analytes. Each LED is paired with a dedicated collimating lens and directed through the optical path using a fixed arrangement in a compact housing, as outlined in the system’s block diagram shown in Figure 1. Light detection is performed via a dual-channel uncooled photodiode setup composed of gallium antimonide (GaSb) and indium gallium arsenide (InGaAs) heterostructures. These semiconductors offer enhanced sensitivity across the NIR spectral range, particularly above 1200 nm, without the need for thermal stabilization—an important feature that contributes to device miniaturization and operational simplicity in clinical settings. The system’s optical resolution is approximately 133 nm at 1000 nm, which reflects the trade-off between spectral detail and portability for point-of-care applications.

Spectral data acquisition was performed in reflectance mode using a 16 µL flow quartz cuvette with a fixed optical path length of 0.2 mm. Each spectrum was obtained by averaging 1000 scans at an integration time of 2.4 ms per scan, resulting in a total measurement time of 2.4 s per sample. This rapid acquisition, coupled with the low-power LED matrix and passive detector architecture, enabled efficient throughput while maintaining an acceptable signal-to-noise ratio for subsequent chemometric processing.

### 2.3. Spectra Preprocessing and Processing Methods

All spectral preprocessing and modeling were carried out with pipelines written in Python 3.13, using the scikit-learn [21], XGBoost [22], and SciPy [23] libraries. The complete analysis scripts are provided in Supplementary File S1 and are also openly available in the project’s GitHub repository; the exact library versions are pinned in the repository environment to ensure full reproducibility. The dataset was split at the formulation level into training (80%; 43 formulations) and independent validation (20%; 11 formulations) sets using a fixed random seed (42). All five FT-NIR or ten miniNIR replicate readings from each formulation were assigned to the same partition. Model selection and hyperparameter optimization were performed only within the training set using nested group-aware cross-validation, with formulation identity used as the grouping variable in the outer and inner folds. Spectral preprocessing, dimensionality reduction and spectral-window selection were fitted within the corresponding training folds. The independent validation set remained untouched until final evaluation. This design prevents replicate leakage and reduces optimistic performance estimates caused by treating repeated readings as independent samples.

Spectra pre-processing included Savitzky–Golay filter [24] followed by the Standard Normal Variate (SNV) transformation [25] and, finally, by standardization of each variable. Due to the significant differences in spectral resolution, for the FT-NIR spectra a 21-point window with a second-order polynomial and first derivative was used, whereas for the six-channel miniNIR data a 5-point window with a second-order polynomial and no derivative (smoothing only) was applied. Principal component analysis (PCA) of the preprocessed validation spectra was used for exploratory data-pattern analysis.

Five regression algorithms were evaluated: Partial Least Squares Regression (PLS), Support Vector Regression (SVR), Random Forest (RF), Gradient Boosting (GB) and Ridge Regression (RR). Hyperparameters were optimized by grid search within the inner cross-validation loop over, among others, the number of PLS latent variables (1–6), the SVR penalty, kernel width and ε-insensitivity, and the number of trees, depth, learning rate and subsampling of the ensemble models; the complete grids are provided in the repository. For the FT-NIR spectra data, models were built on the whole spectrum and, in addition, a sliding spectral-window search was performed to identify the most informative regions, including a window matched to the spectral range of the miniNIR. For the miniNIR spectra data, the six LED channels were used directly. Nested cross-validation (outer 3–5 folds, inner 2–4 folds; exact values in the repository) selected the best model and spectral window per analyte. Candidate preprocessing pipelines and hyperparameters were compared under the same grouped nested cross-validation partitions. The model with the lowest mean outer-fold RMSE was selected without reference to the independent validation results. Performance was reported using R^2^, root mean squared error (RMSE) and mean absolute error (MAE).

For classification, five supervised algorithms were compared under identical formulation-grouped partitions: Support Vector Machine with a radial basis function kernel (SVM-RBF), Random Forest, XGBoost, logistic regression and partial least-squares discriminant analysis (PLS-DA). Binary disease-profile models were selected by mean outer-fold recall, whereas six-class models were selected by mean outer-fold macro-recall. Hyperparameters were selected by grid search within the inner loop (for the SVM, C ∈ {0.1, 1, 10} and γ ∈ {scale, 0.1, 0.01}; for the random forest, 300–600 trees with varying maximum depth, leaf size and feature subsampling; for XGBoost, up to 600 trees with a learning rate of 0.05, maximum depth of 3–5, row and column subsampling of 0.7–0.9, and L2 regularization). As in the regression analysis, the FT-NIR classifiers were evaluated on the whole spectrum, on an optimized spectral window, and on a window matched to the miniNIR range, while the miniNIR used its six channels. Hyperparameter optimization and any decision-threshold tuning were confined to the training folds. Final models were evaluated once on the independent validation formulations using accuracy, precision, recall, F1-score and specificity for binary discrimination, and macro-averaged precision, recall and F1-score for category assignment. ROC-AUC was also reported (binary AUC and one-versus-rest macro- and weighted-AUC for the multiclass task).

## 3. Results and Discussion

### 3.1. Exploratory Analysis by PCA

**Figure 2:**
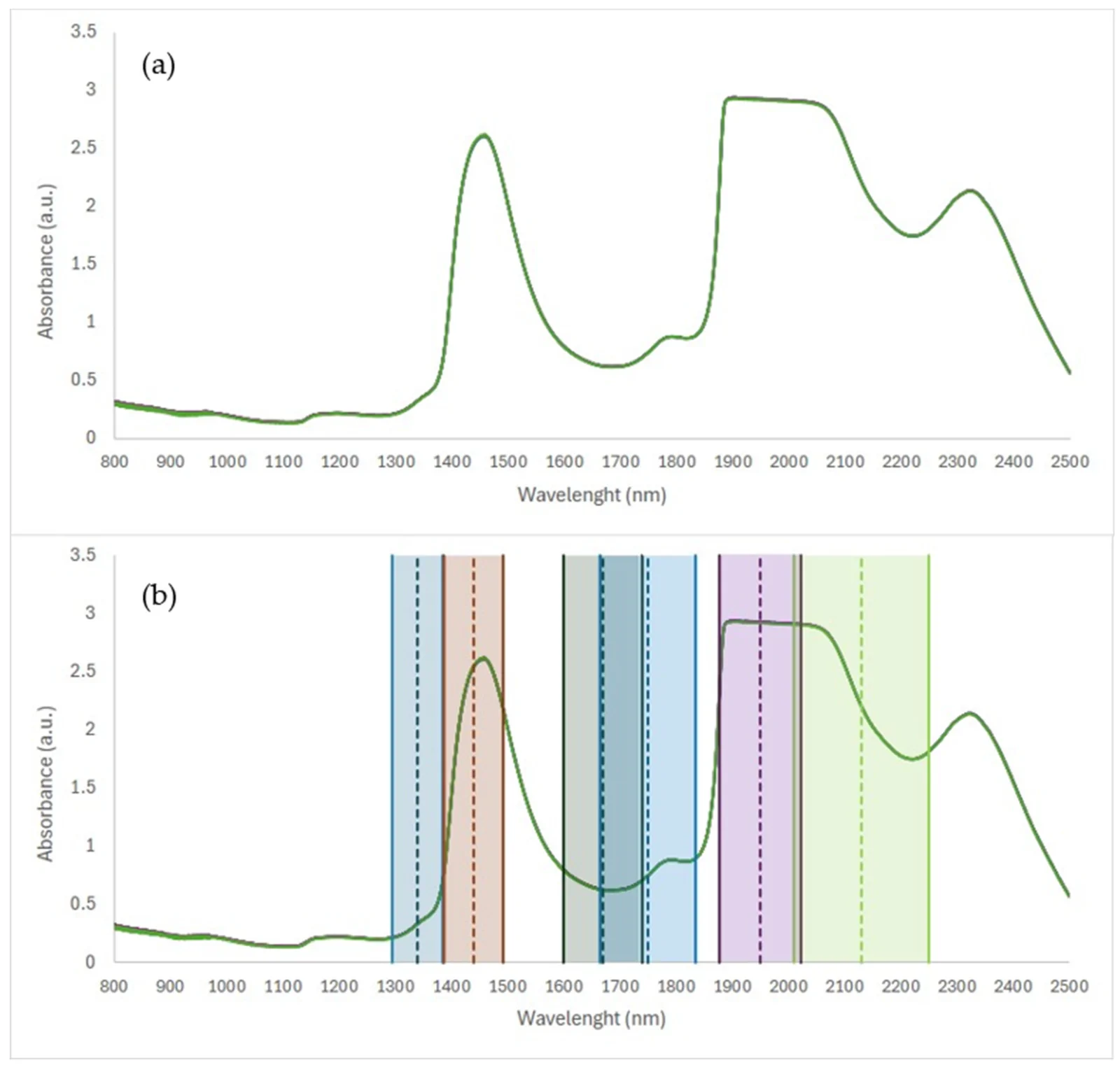
Mean FT-NIR spectra for the six concentration-defined simulated categories and correspondence with the miniNIR LED channels. (**a**) Full FT-NIR range. (**b**) Detail of the miniNIR range with LED spectral coverage. Vertical dashed lines indicate the manufacturer-specified LED peak emissions (1.34, 1.44, 1.67, 1.75, 1.95 and 2.13 µm); shaded regions indicate the manufacturer-specified full width at half maximum (90, 105, 140, 170, 145 and 240 nm, respectively). The overlay illustrates the sparse, broad and partly overlapping sampling of the continuous FT-NIR spectrum; the miniNIR records six channel responses and does not acquire a continuous spectrum.

The 54 serum solutions, spanning imposed healthy-like profiles to concentration patterns corresponding to advanced simulated CKD categories through coordinated variation of creatinine, urea and albumin, were analyzed with both instruments. The FT-NIR provides continuous sampling over 12,500–4000 cm^−1^ (approximately 800–2500 nm), whereas the miniNIR records six broad discrete channels between nominal wavelengths of 1300 and 2100 nm. Figure 2 presents the mean FT-NIR spectrum for each simulated category. The manufacturer-specified LED peak emissions and full widths at half maximum are overlaid on the miniNIR-range panel, making the reduction in spectral sampling explicit.

This configuration was constrained by the LED models commercially available when the prototype was assembled. The covered region includes N–H, O–H and C–H overtone and combination absorptions relevant to creatinine, urea and proteins, but the six channels do not resolve the internal structure of these overlapping bands. In particular, the albumin-informative region identified by the FT-NIR window search is represented by only a small number of broad LED channels.

**Figure 3:**
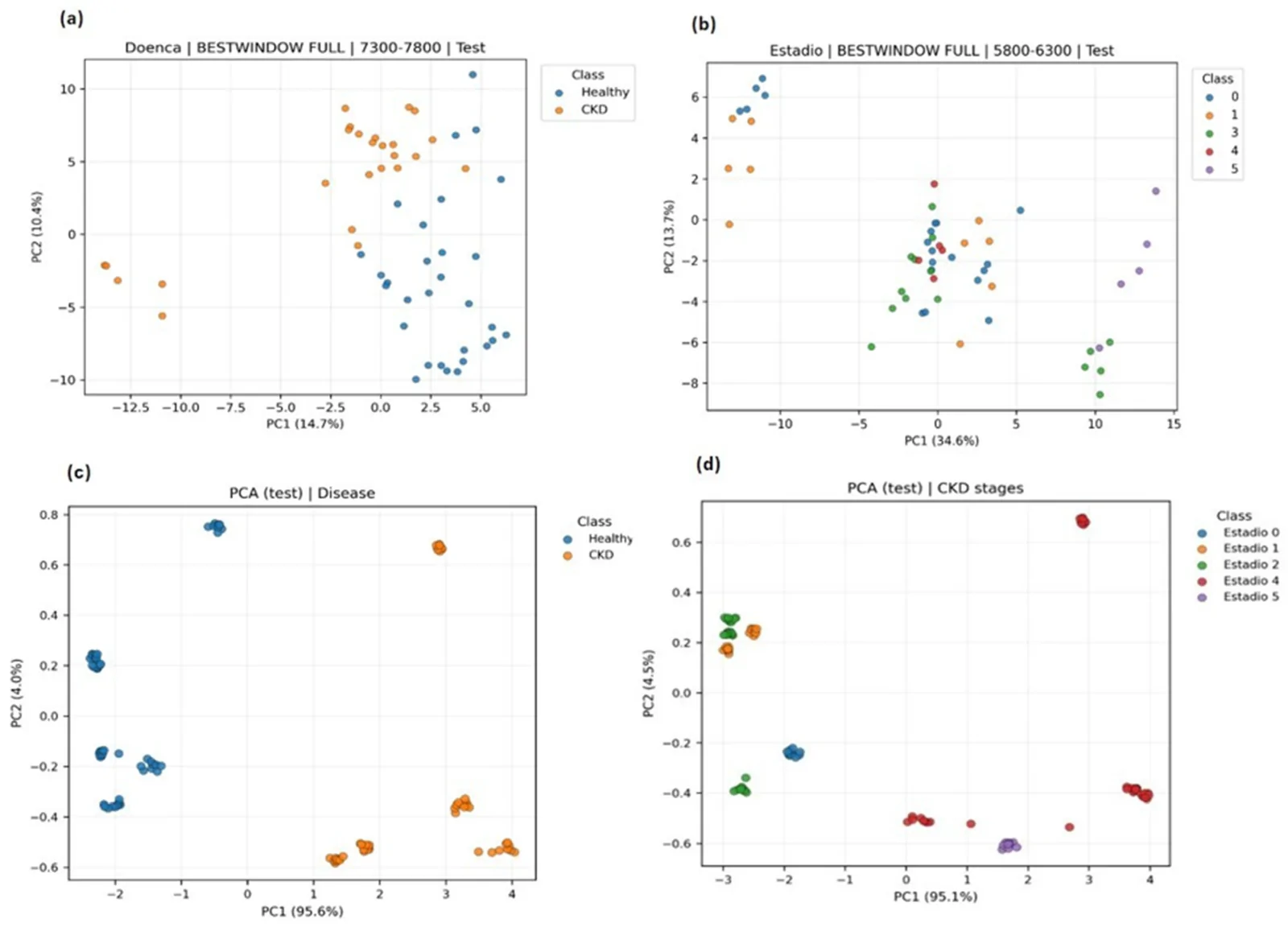
Principal component analysis of the independent validation spectra, colored by disease profile (**a**,**c**) and simulated disease category (**b**,**d**), for the benchtop FT-NIR spectrometer (**a**,**b**) and the portable miniNIR device (**c**,**d**). For each panel, PCA was fitted using the corresponding training data, and the validation spectra were subsequently projected into that space. Axis percentages indicate the variance explained by each principal component.

Exploratory PCA (Figure 3) shows separation associated with the concentration-defined profiles in both platforms. This pattern reflects the coordinated experimental variation of creatinine, urea and albumin. The apparently tighter grouping for miniNIR is also influenced by the low dimensionality and broad, pre-defined channels, whereas the FT-NIR variance is distributed across a much larger number of spectral variables.

### 3.2. Simultaneous Prediction of Creatinine, Urea, and Albumin

**Figure 4:**
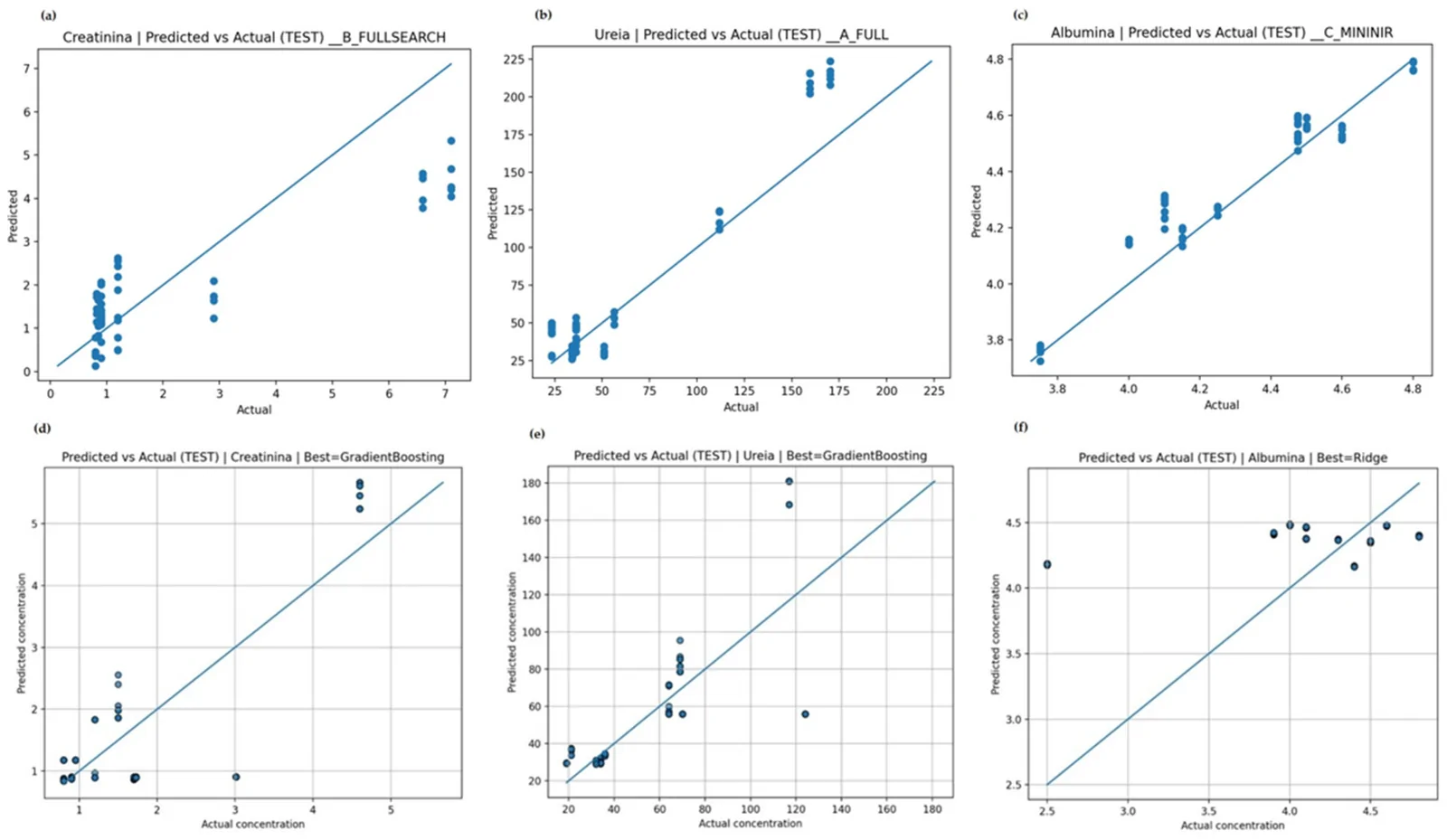
Predicted versus reference concentrations (validation set) for serum creatinine (**a**,**d**), urea (**b**,**e**), and albumin (**c**,**f**), obtained with the benchtop FT-NIR spectrometer (**a**–**c**) and with the portable miniNIR device (**d**–**f**).

Regression models were developed for each analyte from the FT-NIR spectra (whole spectrum and after spectral-window optimization) and from the six miniNIR bands (Table 2, Figure 4). For FT-NIR, the best validation results among the reported spectral configurations were obtained for albumin with PLS in the 5632–6500 cm^−1^ window (R^2^ = 0.89; RMSE = 0.10), urea with Gradient Boosting over the full spectrum (R^2^ = 0.79; RMSE = 17.4), and creatinine with Random Forest in the 7800–8400 cm^−1^ window (R^2^ = 0.64; RMSE = 1.36). Full-spectrum albumin prediction did not generalize (R^2^ = −0.04), illustrating the importance of narrowing the spectroscopical window. This observation was not so evident for urea (R^2^ = 0.79 for full length vs R^2^ = 0.78 for 5400–6000 cm^−1^ window) and for creatinine (R^2^ = 0.60 for full length vs R^2^ = 0.64 for 7800–8400 cm^−1^ window).

For miniNIR, the models selected by grouped nested cross-validation yielded validation R^2^ values of 0.48 for creatinine (Gradient Boosting; RMSE = 0.82), 0.30 for urea (Gradient Boosting; RMSE = 29.1), and −0.10 for albumin (Ridge; RMSE = 0.61). Thus, the portable device retained moderate analytical information for creatinine and urea in this controlled dataset, whereas albumin prediction did not generalize to the held-out formulations. The predicted-versus-reference plots (Figure 4) illustrate this pattern: a reasonable alignment along the identity line for both instruments for creatinine and urea, and a clear loss of agreement for albumin when moving from the benchtop spectrometer to the portable device.

### 3.3. Supervised Disease Classification

**Figure 5:**
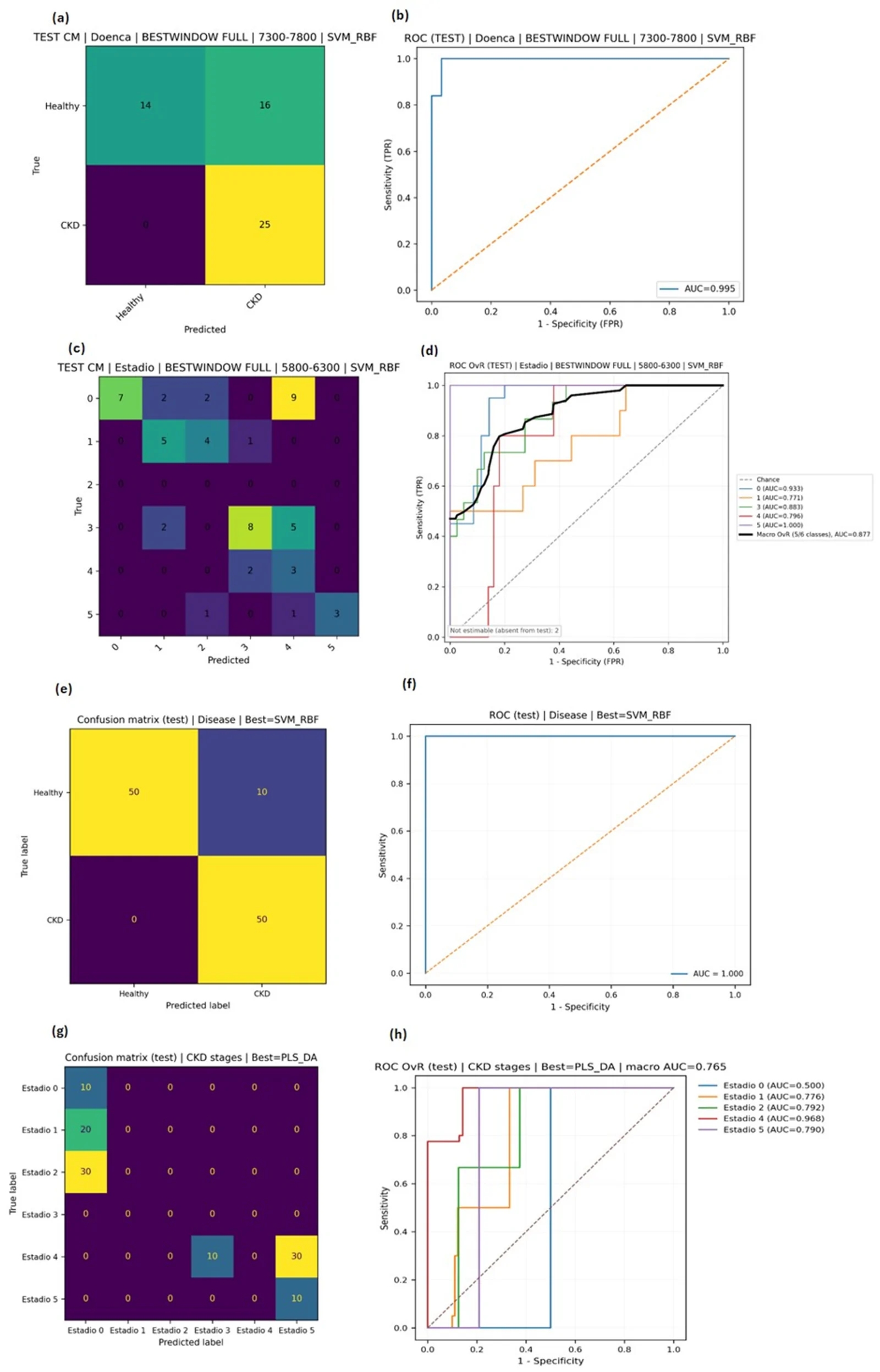
Classification results (validation set) for the benchtop FT-NIR spectrometer (top row, (**a**–**d**)) and the portable miniNIR device (bottom row, (**e**–**h**)). For each instrument: confusion matrix (**a**,**e**) and ROC curve (**b**,**f**) for disease detection, and confusion matrix (**c**,**g**) and macro-averaged one-versus-rest ROC curve (**d**,**h**) for simulated CKD-category assignment. FT-NIR panels correspond to the optimized spectral window; miniNIR panels use the six LED bands. Multiclass ROC curves comprise the five categories represented in the validation set.

Supervised classifiers were trained to distinguish healthy from diseased profiles and to assign the six simulated CKD categories (Table 3, Figure 5). For binary discrimination of healthy/CKD-like formulations, model selection was based exclusively on grouped nested-cross-validation recall in the training set. On the held-out formulations, miniNIR SVM-RBF achieved AUC = 1.00, accuracy = 0.91, recall = 1.00 and specificity = 0.83. FT-NIR results depended strongly on spectral configuration: the optimized 7300–7800 cm^−1^ window achieved AUC = 0.99, accuracy = 0.71 and specificity = 0.47.

The six-class assignment was considerably less robust. For FT-NIR, Random Forest was selected as the best model for the full spectrum, SVM-RBF for the optimized 5800–6300 cm^−1^ window, and SVM-RBF for the miniNIR-matched 5732–6032 cm^−1^ window. Their validation accuracies were 0.47, 0.47 and 0.38, respectively, with macro-AUC values of 0.90, 0.91 and 0.87 across the classes. For miniNIR, PLS-DA was selected by training macro-recall, but validation accuracy was 0.18 and macro-F1 was 0.11; macro-AUC was 0.77 across the classes. These results do not support reliable category assignment in the present small dataset.

Overall, the six-channel device retained enough information to separate the imposed binary concentration profiles in this held-out set, but it did not support reliable six-category assignment and provided limited regression performance, particularly for albumin. The benchtop FT-NIR supplied broader continuous spectral information and generally stronger regression and category-assignment results. Accordingly, the present findings define the analytical losses associated with six-channel sampling and motivate further hardware optimization; they do not establish clinical complementarity, CKD-specific recognition or readiness for point-of-care screening.

## 4. Conclusions

This study provides an analytical comparison of miniaturized and benchtop near-infrared spectroscopy in 54 composed serum formulations. MiniNIR achieved promising binary discrimination of the imposed healthy-like and CKD-like concentration profiles, but showed only moderate creatinine and urea regression, no generalizable albumin regression, and weak six-class assignment. FT-NIR generally provided stronger regression results, with both regression and classification performances varying with spectral window.

Because the disease labels were constructed by coordinated variation of three analytes in a single FBS matrix, the models may identify the imposed concentration structure rather than CKD-specific biology. Larger independent studies using human serum, clinically defined outcomes and external validation are required before any screening or staging application can be considered.

The use of fetal bovine serum rather than human samples is an important limitation, since species differences and the absence of patient-level biological and pre-analytical variability may affect spectral signatures and model generalizability. In addition, the 54 formulations were generated by coordinated variation of creatinine, urea and albumin; consequently, classification may recover the designed concentration structure rather than disease-specific biology. Although formulation-grouped nested cross-validation and an untouched 11-formulation validation set reduce replicate leakage, this small validation set provides limited evidence of generalizability and did not contain all six simulated categories. Future work should use larger, independent and clinically characterized human cohorts, evaluate relevant confounders and include external validation.

The FT-NIR results obtained within spectral windows accessible to the portable device provide additional guidance for hardware optimization. Within these regions, FT-NIR achieved higher validation performance than the six-channel miniNIR for creatinine, urea and albumin regression, as well as for simulated-category assignment. This indicates that analytically relevant information is present within the spectral range already covered by the prototype, but may not be fully captured by six relatively broad and widely spaced LED channels. Increasing channel density at selected wavelengths could improve the representation of local band shapes and overlapping absorptions while retaining the advantages of an LED-based architecture. Future development should therefore use the FT-NIR window-selection results as a starting point for systematic wavelength selection, followed by experimental validation of progressively denser LED configurations. This would allow identification of the minimum number and optimal distribution of channels required to approach FT-NIR performance without compromising cost, acquisition speed and portability.

Despite these limitations, miniNIR demonstrates practical advantages in portability, speed and cost. The present data justify further analytical and clinical validation, but not the selection of the device for CKD screening or staging at this stage.

## Figures and Tables

**Table 1:** Some recent spectroscopic approaches for renal-biomarker analysis.

Study	Technique/Platform	Portable	Matrix	Target(s)	Main Outcome
Suzuki et al., 2018 [16]	LED-based NIR (discrete diodes)	Yes	Spot urine	Urea, creatinine (U/C ratio)	Reagentless estimation feasible
Mengyao Chen et al., 2024 [19]	Lightweight NIR	Yes (UrineSpec)	Urine	Metabolites	Low-cost metabolite detection
Tangwanichgapong et al., 2025 [6]	ATR-FTMIR	Benchtop	Saliva/serum	CKD screening biomarkers	Discrimination of CKD
Hall & Pollard, 1993 [8]	NIR	Benchtop	Serum	Total protein, albumin, urea	Reagent-free serum quantification
Barnea & Abookasis, 2019 [9]	NIR	Benchtop	Blood	Creatinine	High agreement vs. assay
Serrano et al., 2025 [10]	FT-NIR & FT-MIR	Benchtop	Serum	Creatinine, urea, albumin	R^2^ > 0.9 (regression)
Serrano et al., 2026 [11]	FT-NIR & FT-MIR	Benchtop	Spiked serum (simulated stages)	Biomarkers; CKD staging (KDIGO stages)	Stage classification
This work	LED-based NIR (6 LEDs) vs. benchtop FT-NIR	Yes	Spiked serum (simulated CKD stages)	Biomarkers; disease & CKD staging (KDIGO stages)	Portable vs. benchtop benchmark

**Table 2:** Regression performance (validation set) for serum creatinine, urea, and albumin obtained with the benchtop FT-NIR spectrometer (whole spectrum and optimized spectral window) and with the portable miniNIR device. R^2^ (CV) is the nested cross-validation coefficient of determination. RMSE units are mg/dL for creatinine and urea and g/dL for albumin. The best model per analyte is highlighted in bold.

Analyte	Platform/ Spectral Range	Model	Calibration	Validation	
			R^2^ (CV)	R^2^	RMSE
**Creatinine**	FT-NIR full spectrum	PLS	0.75	0.60	1.43
	**FT-NIR optimized window (7800–8400 cm^−1^)**	**RF**	**0.63**	**0.64**	**1.36**
	FT-NIR miniNIR window (5632–6500 cm^−1^)	PLS	0.68	0.64	1.37
	miniNIR (4762–7692 **cm^−1^)**	GB	0.68	0.48	0.82
**Urea**	**FT-NIR full spectrum**	**GB**	**0.79**	**0.79**	**23.8**
	FT-NIR optimized window (5400–6000 cm^−1^)	RF	0.64	0.78	24.4
	FT-NIR miniNIR window (4512–5513 cm^−1^)	Ridge	0.63	0.68	29.4
	miniNIR (4762–7692 cm^−1^)	GB	0.67	0.30	29.1
Albumin	FT-NIR full spectrum	GB	0.16	−0.04	0.30
	FT-NIR optimized window (5700–6300 cm^−1^)	PLS	0.87	0.87	0.10
	**FT-NIR miniNIR window** **(5632–6500 cm^−1^)**	**PLS**	**0.13**	**0.89**	**0.10**
	miniNIR (4762–7692 cm^−1^)	Ridge	−0.18	−0.10	0.61

**Table 3:** Classification performance (validation set) for disease detection (healthy vs. diseased) and simulated CKD-category assignment, obtained with the benchtop FT-NIR spectrometer (whole spectrum, optimized window, and benchtop spectrum restricted to the miniNIR spectral range) and with the portable miniNIR device (6 LEDs, 1300–2100 nm). The best model per task is highlighted in bold.

Task	Platform/ Spectral Range	Model	AUC	Accuracy	Precision	Recall	F1	Specificity
**SimulatedDisease profile**	FT-NIR full spectrum	RF	0.93	0.45	0.45	1.00	0.63	0.00
	**FT-NIR optimized window** **(7300–7800 cm^−1^)**	**SVM RBF**	**0.99**	**0.71**	**0.61**	**1.00**	**0.76**	**0.47**
	FT-NIR miniNIR window (5632**–**6500 cm^−1^)	SVM RBF	0.00	0.45	0.45	1.00	0.63	0.00
	miniNIR (4762**–**7692 cm^−1^)	SVM-RBF	1.00	0.91	0.83	1.00	0.91	0.83
**Simulated category**	FT-NIR full spectrum	RF	0.86	0.47	0.51	0.45	0.43	—
	**FT-NIR optimized window** **(5800–6300 cm^−1^)**	**SVM RBF**	**0.88**	**0.47**	**0.57**	**0.43**	**0.44**	**—**
	FT-NIR miniNIR-matched window (5732**–**6032 cm^−1^)	SVM RBF	0.87	0.38	0.46	0.33	0.36	—
	miniNIR (4762**–**7692 cm^−1^)	PLS-DA	0.77	0.18	0.07	0.33	0.11	—

## Data Availability

The data presented in this study will be available upon request from the corresponding author.

## Supplementary Materials

The following supporting information can be downloaded at: https://github.com/PJZoio/NIRU-and-NIR-Classification-and-Regression-algorithms (accessed on 12 May 2026).

## References

[1] Baker M.J., Trevisan J., Bassan P., Bhargava R., Butler H.J., Dorling K.M., Fielden P.R., Fogarty S.W., Fullwood N.J., Heys K.A. (2014). Using Fourier Transform IR Spectroscopy to Analyze Biological Materials. Nat. Protoc..

[2] Delrue C., De Bruyne S., Speeckaert M.M. (2023). The Potential Use of Near- and Mid-Infrared Spectroscopy in Kidney Diseases. Int. J. Mol. Sci..

[3] Beć K.B., Grabska J., Huck C.W. (2020). Near-Infrared Spectroscopy in Bio-Applications. Molecules.

[4] Gullifa G., Barone L., Papa E., Giuffrida A., Materazzi S., Risoluti R. (2023). Portable NIR Spectroscopy: The Route to Green Analytical Chemistry. Front. Chem..

[5] Tangwanichgapong K., Klanrit P., Chatchawal P., Wongwattanakul M., Pongskul C., Chaichit R., Hormdee D. (2025). Identification of Molecular Biomarkers in Human Serum for Chronic Kidney Disease Using ATR-FTIR Spectroscopy. Spectrochim. Acta A Mol. Biomol. Spectrosc..

[6] Tangwanichgapong K., Klanrit P., Chatchawal P., Wongwattanakul M., Pongskul C., Chaichit R., Hormdee D. (2025). Salivary Attenuated Total Reflectance–Fourier Transform Infrared Spectroscopy Combined with Chemometric Analysis: A Potential Point-of-Care Approach for Chronic Kidney Disease Screening. Photodiagnosis Photodyn. Ther..

[7] Jiménez-Ruiz J.A., Reinoso-Arija R., Marin-Hinojosa C., Fernandez-Boza A., Muñoz-Sanchez B., Carrera-Cueva C., Caballero C., Quintana-Gallego E., Otero-Candelera R., Lopez-Campos J.L. (2025). Evaluation of a Near Infrared Spectroscopy Based Method for the Estimation of Substance P in Saliva of Patients with COPD. Sci. Rep..

[8] Hall J.W., Pollard A. (1993). Near-Infrared Spectroscopic Determination of Serum Total Proteins, Albumin, Globulins, and Urea. Clin. Biochem..

[9] Barnea Z.H., Abookasis D. (2019). Determination of Creatinine Level in Patient Blood Samples by Fourier NIR Spectroscopy and Multivariate Analysis. J. Innov. Opt. Health Sci..

[10] Serrano D., Zoio P., Fonseca L.P., Calado C.R.C. (2025). Comparison of Mid and Near-Infrared Spectroscopy to Predict Creatinine, Urea and Albumin in Serum Samples as Biomarkers of Renal Function. Biosensors.

[11] Serrano D., Zoio P., Fonseca L.P., Calado C.R.C. (2026). Prediction of Chronic Kidney Disease Based on Simulated Serum Analysis by Vibrational Spectroscopy. Biosensors.

[12] Crocombe R.A. (2018). Portable Spectroscopy. Appl. Spectrosc..

[13] Bertinetto C.G., Schoot M., Dingemans M., Meeuwsen W., Buydens L.M.C., Jansen J.J. (2022). Influence of Measurement Procedure on the Use of a Handheld NIR Spectrophotometer. Food Res. Int..

[14] van Kollenburg G.H., van Manen H.-J., Admiraal N., Gerretzen J., Jansen J.J. (2021). Low-Cost Handheld NIR Spectroscopy for Identification of Organic Solvents and Low-Level Quantification of Water Contamination. Talanta.

[15] Premasinghe C.N., Broeckelmann C., Kolobaric A., Cozzolino D., Chapman J. (2026). Artificial Intelligence-Enhanced Near Infrared (NIR) Spectroscopy for Rapid Diagnostics of Infectious Diseases. Appl. Spectrosc. Rev..

[16] Suzuki I., Ogawa M., Seino K., Nogawa M., Naito H., Yamakoshi K.I., Tanaka S. (2018). Reagentless Estimation of Urea and Creatinine Concentrations Using Near-Infrared Spectroscopy for Spot Urine Test of Urea-to-Creatinine Ratio. Adv. Biomed. Eng..

[17] Shaw R.A., Kotowich S., Mantsch H.H., Leroux M. (1996). Quantitation of Protein, Creatinine, and Urea in Urine by Near-Infrared Spectroscopy. Clin. Biochem..

[18] Pezzaniti J.L., Jeng T.W., McDowell L., Oosta G.M. (2001). Preliminary Investigation of Near-Infrared Spectroscopic Measurements of Urea, Creatinine, Glucose, Protein, and Ketone in Urine. Clin. Biochem..

[19] Chen M., Chen H., Niu S. (2024). UrineSpec: A Lightweight Near-Infrared Spectroscopy System for Metabolite Detection in Urine. Proceedings of the 22nd ACM Conference on Embedded Networked Sensor Systems (SenSys ‘24), Hangzhou, China, 4–7 November 2024.

[20] Monteiro L.L., Zoio P., Carvalho B.B., Fonseca L.P., Calado C.R.C. (2023). Quality Monitoring of Biodiesel and Diesel/Biodiesel Blends: A Comparison between Benchtop FT-NIR versus a Portable Miniaturized NIR Spectroscopic Analysis. Processes.

[21] Pedregosa F., Varoquaux G., Gramfort A., Michel V., Thirion B., Grisel O., Blondel M., Prettenhofer P., Weiss R., Dubourg V. (2011). Scikit-Learn: Machine Learning in Python. J. Mach. Learn. Res..

[22] Chen T., Guestrin C. (2016). XGBoost: A Scalable Tree Boosting System. Proceedings of the 22nd ACM SIGKDD International Conference on Knowledge Discovery and Data Mining, San Francisco, CA, USA, 13–17 August 2016.

[23] Virtanen P., Gommers R., Oliphant T.E., Haberland M., Reddy T., Cournapeau D., Burovski E., Peterson P., Weckesser W., Bright J. (2020). SciPy 1.0: Fundamental Algorithms for Scientific Computing in Python. Nat. Methods.

[24] Savitzky A., Golay M.J.E. (1964). Smoothing and Differentiation of Data by Simplified Least Squares Procedures. Anal. Chem..

[25] Barnes R.J., Dhanoa M.S., Lister S.J. (1989). Standard Normal Variate Transformation and De-Trending of Near-Infrared Diffuse Reflectance Spectra. Appl. Spectrosc..

